# Integrated transcriptomic analysis identifies MZT2A as a prognostic and immune-related biomarker in KIRC

**DOI:** 10.1097/JS9.0000000000003062

**Published:** 2025-07-15

**Authors:** Yunjing Zhang, Zekun Xin, Xinnuo Li, Yanmei Chen

**Affiliations:** a Department of Anesthesiology, Hebei Petro China Central Hospital, Langfang, China; b Department of Urology, Hebei Petro China Central Hospital, Langfang, China

**Keywords:** KIRC, MZT2A, prognostic biomarker, tumor microenvironment

## Abstract

Renal cell carcinoma (RCC) represents the most common solid renal malignancy, accounting for 90% of kidney cancers and 3% of all malignancies. Clear cell RCC (ccRCC) is the predominant histologic subtype, comprising roughly 80% of cases. Despite a 5-year survival rate of 75%, up to 30% of patients with localized ccRCC eventually develop recurrence or distant metastases. Mitotic spindle organizing protein 2A (MZT2A), also known as FAM128A, GCP8, or MOZART2A, is located on chromosome 2q21.1 and encodes a 158-amino acid protein first described by Hutchins et al. in 2010. To date, limited studies have linked MZT2A to tumorigenesis, primarily in lung and gastric cancers. The tumor microenvironment (TME), particularly tumor-infiltrating immune cells, plays a critical role in cancer progression and prognosis. Using paired tumor and normal samples from TCGA, we assessed MZT2A expression across multiple cancer types. Collectively, these findings suggest that MZT2A is involved in immune cell infiltration and the tumor microenvironment, may promote KIRC progression, and is overexpressed in cases with poor outcomes, supporting its potential as a diagnostic and prognostic biomarker.

*Dear Editor,*

Renal cell carcinoma (RCC) is the most common solid malignancy of the kidney, accounting for approximately 90% of all kidney cancers and 3% of all malignancies^[1]^. Clear cell renal cell carcinoma (ccRCC) is the predominant histologic subtype, accounting for approximately 80% of all RCC cases^[2]^. Although the 5-year survival rate for localized ccRCC is 75%, recurrence or distant metastasis occurs in up to 30% of cases^[3]^. Accordingly, the identification of reliable biomarkers for early detection and prognostication remains a critical clinical imperative. This manuscript is conducted in accordance with the TITAN Guidelines 2025^[4]^.

Mitotic spindle organizing protein 2A (MZT2A), also known as FAM128A, GCP8, or MOZART2A, is located on chromosome 2q21.1 and encodes a 158-amino acid protein first described by Hutchins *et al* in 2010^[5]^. Current evidence linking MZT2A to tumorigenesis is limited and has mainly focused on lung and gastric cancers^[6]^.

The tumor microenvironment (TME), particularly tumor-infiltrating immune cells, plays a pivotal role in cancer progression and prognosis. In this study, we analyzed MZT2A expression across multiple cancer types using paired tumor and normal tissue samples from The Cancer Genome Atlas (TCGA). Our objective was to investigate the association between MZT2A expression and patient outcomes, as well as to explore its potential involvement in tumor biology and the immune microenvironment.

MZT2A expression was significantly upregulated in KIRC tissues compared to normal controls, as demonstrated by TCGA data (Fig. 1A-C) and corroborated by protein-level validation from the HPA database (Fig. 1D-E). Elevated MZT2A expression was positively correlated with T stage, M stage, and pathological grade (Fig. 1F-H), as well as with relevant clinical parameters detailed in Table 1. Patients with elevated MZT2A expression showed significantly reduced overall survival (*P* < 0.05), suggesting its association with worse prognosis (Fig. 1I). ROC analysis demonstrated robust diagnostic accuracy, with an AUC of 0.923 (Fig. 1J). Gene Ontology (GO) and Kyoto Encyclopedia of Genes and Genomes (KEGG) analyses indicated MZT2A may promote KIRC progression through pathways related to regulation of pH, keratinization, monovalent inorganic cation homeostasis, collecting duct acid secretion, synaptic vesicle cycle, neuroactive ligand-receptor interaction, retinol metabolism, and drug metabolism (Fig. 1K-L). TISIDB analysis further revealed strong correlations between MZT2A expression and various immune regulators, including immunostimulators, immunoinhibitors, chemokines, and receptors (Fig. 1M-P). Multiple immune deconvolution algorithms (CIBERSORT, EPIC, MCPcounter, quanTIseq, and TIMER) consistently associated MZT2A with immune cell infiltration (Fig. 1Q-U). Single-cell RNA-seq datasets (GSE111360, GSE121636, GSE139555, GSE159115) confirmed MZT2A expression in epithelial cells, malignant cells, regulatory T cells, and various other T-cell subsets (Fig. 1V-Y).

**Figure 1.**
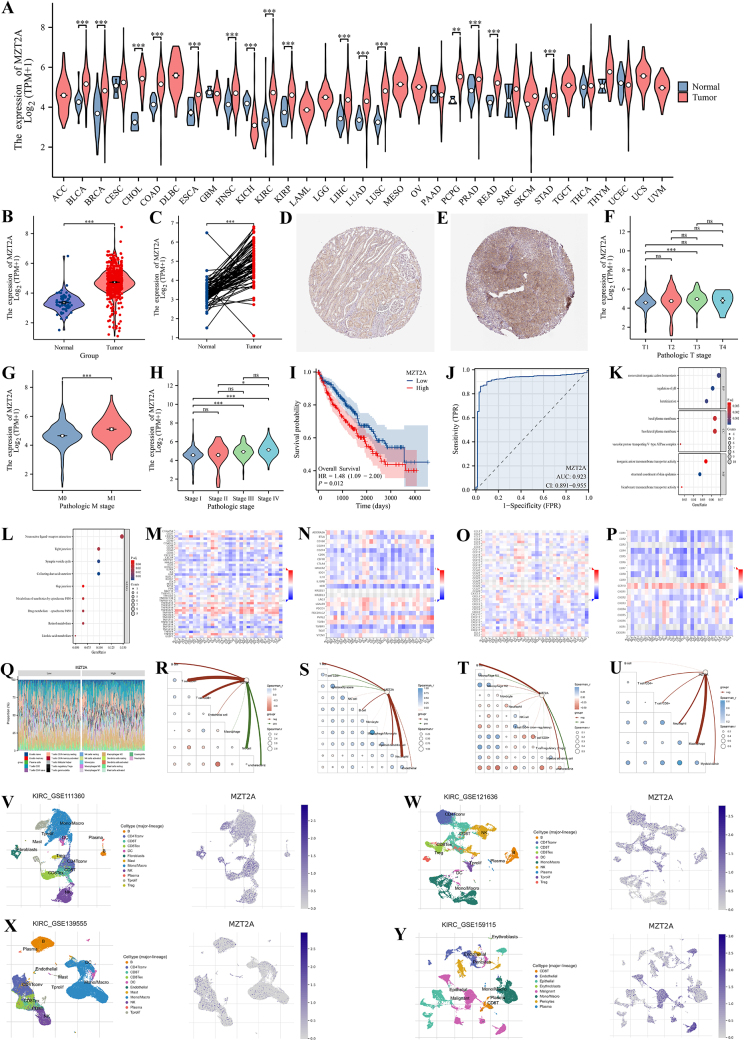
(A) MZT2A expression across tumor and normal tissues in multiple cancer types based on TCGA data. (B-C) Differential expression of MZT2A in unpaired and paired KIRC samples. (D-E) Immunohistochemical validation of elevated MZT2A protein levels in KIRC from the HPA database. (F-H) Correlation between MZT2A expression and T stage, M stage, and pathologic grade. (I) Kaplan-Meier analysis of overall survival stratified by MZT2A expression. (J) ROC curve assessing the diagnostic accuracy of MZT2A. (K-L) GO and KEGG enrichment analyses of MZT2A-associated differentially expressed genes in KIRC. (M-Y) Analysis of immune landscape in relation to MZT2A expression, including TISIDB correlations, immune infiltration scores, and single-cell RNA-seq data.

**Table 1:** Association between MZT2A expression and clinicopathological features in TCGA-KIRC

Characteristics	Low expression of MZT2A	High expression of MZT2A	*P* value
*n*	270	271	
Pathologic T stage, *n* (%)			<0.001
T1	166 (30.7%)	113 (20.9%)	
T2	30 (5.5%)	41 (7.6%)	
T3	69 (12.8%)	111 (20.5%)	
T4	5 (0.9%)	6 (1.1%)	
Pathologic N stage, *n* (%)			0.081
N0	130 (50.4%)	112 (43.4%)	
N1	5 (1.9%)	11 (4.3%)	
Pathologic M stage, *n* (%)			0.002
M0	229 (45.1%)	200 (39.4%)	
M1	27 (5.3%)	52 (10.2%)	
Pathologic stage, *n* (%)			< 0.001
Stage I	165 (30.7%)	108 (20.1%)	
Stage II	28 (5.2%)	31 (5.8%)	
Stage III	47 (8.7%)	76 (14.1%)	
Stage IV	27 (5%)	56 (10.4%)	
Gender, *n* (%)			0.080
Female	103 (19%)	84 (15.5%)	
Male	167 (30.9%)	187 (34.6%)	
Age, *n* (%)			0.094
≤ 60	144 (26.6%)	125 (23.1%)	
> 60	126 (23.3%)	146 (27%)	

Collectively, these findings suggest that MZT2A is involved in immune cell infiltration and the tumor microenvironment, may contribute to KIRC progression, and is overexpressed in cases with poor prognosis, supporting its potential as a diagnostic and prognostic biomarker.

## Data Availability

All data are obtained in the article.
